# Association of VEGFA polymorphisms with susceptibility and clinical outcome of hepatocellular carcinoma in a Chinese Han population

**DOI:** 10.18632/oncotarget.14870

**Published:** 2017-01-27

**Authors:** Fei Liu, Limei Luo, Yonggang Wei, Wentao Wang, Tianfu Wen, Jiayin Yang, Mingqing Xu, Bo Li

**Affiliations:** ^1^ Department of Liver Surgery and Liver Transplantation Center, West China Hospital, Sichuan University, Chengdu, 610041, Sichuan Province, China; ^2^ Department of Clinical Immunological Laboratory, West China Hospital, Sichuan University, 610041, China

**Keywords:** hepatocellular carcinoma, VEGFA, polymorphism, susceptibility, clinical outcome

## Abstract

Vascular endothelial growth factor A (VEGFA) is an important angiogenesis regulator, which plays an important role in angiogenesis and progression of tumor, including hepatocellular carcinoma (HCC). We aimed at determining whether single nucleotide polymorphisms of VEGFA gene influence the development and clinical outcomes of HCC. We analyzed four potential functional polymorphisms (936C/T, 634G/C, 1612G/A, 2578C/A) of VEGFA gene in 476 HCC patients and 526 controls using matrix-assisted laser desorption ionization time-of-flight mass spectrometry method. Serum VEGF levels were measured by enzyme-linked immunosorbent assay. The Kaplan-Meier methods with log-rank test and Cox regression models were used to compare survival of resected HCC patients according to the genotype. We found that only the VEGFA 2578C/A polymorphism was significantly associated with decreased risk of HCC (AA/AC vs. CC; adjusted OR = 0.69, 95% CI = 0.51−0.93). Furthermore, the 2578C/A polymorphism was associated with significantly decreased postoperative recurrence (AA/AC vs. CC, adjusted OR = 0.51; 95% CI, 0.29−0.88) and improved overall survival (AA/AC vs. CC, adjusted HR = 0.27, 95% CI = 0.13−0.52) of resected HCC patients. In addition, the VEGF serum levels in HCC patients were significantly higher than those in healthy controls, although no significant association between VEGFA genotype and serum levels of VEGF was observed. These results suggest that the VEGFA 2578 C/A polymorphism may play a potential role in the development and clinical outcome of HCC among Chinese Han population.

## INTRODUCTION

Liver cancer in men is the fifth most common cancer and the second-leading cause of cancer-related death worldwide [1]. Among primary liver cancers, hepatocellular carcinoma (HCC) is responsible for 70% −90% of the total liver cancer burden worldwide [2]. The pathogenesis of HCC is not completely understood, but it is thought to be a multistage process with the complex interactions of various risk factors including infection with hepatitis B virus (HBV) and/or hepatitis C virus (HCV), cirrhosis, male gender, concurrent alcohol use, aflatoxin B1 intake and multiple genetic variants [3–5]. However, only a fraction of HBsAg carriers eventually develop HCC and only 2.5% of HCV infected individuals develop HCC later in life [6]. Moreover, the variability in outcome following the same environmental exposure and the clustering of HCC within families were observed [7–10], which suggested that host genetic factors may affect HCC development.

Angiogenesis, which defined as the formation of new blood vessels from existing vasculature, represents an essential process in the pathogenesis of cancer, since newly formed vessels promote tumor growth by supplying oxygen and nutrients. HCC is a typical hypervascular tumor, and a radiology finding of an arterial hypervascular pattern is a diagnostic criterion for HCC [11].Vascular endothelial growth factor (VEGF) is a major driver of physiological and pathological angiogenesis [12]. VEGF plays an important role in tumor angiogenesis through promoting endothelial cell growth and migration [13]. VEGF is expressed by several tumors at higher levels than when it appears in normal tissues, and its over-expression suggests unfavorable prognosis [14, 15]. The expression levels of VEGFA mRNA in HCC was 6.95-fold higher than in HBsAg-negative healthy individuals [15]. The serum concentrations of VEGF-A have been found elevated in HCC in parallel with the tumoral grading, and they are considered independent markers of prognosis and survival [16].

The human VEGF gene is located on chromosome 6p21.3 [17] which is highly polymorphic. To date, it has been reported that there are at least 20 potential functional single nucleotide polymorphisms (SNPs) in VEGF gene. Among them, -2578C>A (rs699947), -634G>C (rs2010963), -1612 G/A (rs10434), and -936C>T (rs3025039) were demonstrated to regulate VEGF expression [18, 19]. Accordingly, these polymorphisms may affect VEGF protein concentrations influencing the angiogenesis process and may be related to inter individual variations in tumor risk, progression, and prognosis. In recent years, the VEGF SNPs have been reported to be associated with cancers of the lung [20], colorectum [21], breast, and bladder [22–23]. Moreover, the VEGF SNPs have also been reported to be associated with clinical outcome of esophageal cancer, renal cell carcinoma and non-small cell lung cancer [24–26].

Till date, the association between VEGF SNPs and HCC risk is still controversial and ambiguous [15, 27–28]. Meanwhile, the association between VEGF SNPs and HCC prognosis is also obscure [29–31]. Moreover, no study had investigated the association of VEGF SNPs with risk and clinical prognosis of HCC simultaneously in the same cohort patients. Therefore, we performed a study to comprehensively evaluate the association of VEGF -936C>T, -634G>C, -1612 G/A and -2578C>A polymorphisms with susceptibility and clinical outcome of HCC in a Chinese Han population.

## RESULTS

### Characteristics of studies population

The baseline characteristics of the 476 HCC cases and 526 controls are summarized in Supplementary Table 3. No significant differences between the two groups in terms of age, gender,drinking status and family history of cancer distribution. However, as shown in Supplementary Table 3, there was significant difference between cases and controls in the distribution of smoking and HBV carrier state. The HCC patient characteristics and clinical features of the tumor are summarized in Supplementary Table 4. Of the 476 patients, 222 had their primary tumor surgically resected; the remaining 254 patients underwent local therapy such as transarterial embolization, radiofrequency ablation, or conservative management. We followed HCC patients to December 2012 and the median follow-up period was 22 months (range: 3–62 months).

### Genotypes frequency and effects on the development of HCC

The genotype distributions of VEGF -936C>T, -634G>C, -1612 G/A and -2578 C>A polymorphisms in the cases and the controls were shown in Table 1. The distributions of these genotype frequencies in controls were all in HWE (*P* = 0.54, 0.94, 0.88 and 0.88 for -936C>T, -634G>C, -1612 G/A and -2578 C>A, respectively). In the overall analysis, we found that only the -2578 C>A was significantly associated with the development of HCC. Supplementary Figure 1 shows the three genotypes for VEGF -2578 C>A polymorphism. Compared with the CC genotype, the variant AA and the CA genotypes significantly decreased the risk of developing HCC; the adjusted OR was 0.51 (95% CI = 0.28–0.92) and 0.75 (95% CI = 0.58–0.98), respectively (Table 1).

**Table 1:** VEGFA genotype and allele frequencies of the cases and controls and their association with risk of HCC

Polymorphism	Genotype/allele	HCC (*n* = 476)	controls (*n* = 526)	Crude OR 95% CI	*P*^a^ value	Adjusted OR^b^ 95% CI	*P*^c^ value
VEGF	CC	359 (75.4)^d^	370 (70.3)	1.00		1.00	
936 C/T	CT	112 (23.5)	140 (26.6)	0.83 [0.62,1.10]	0.188	0.84 [0.60,1.19]	0.325
	TT	5 (1.1)	16 (3.1)	**0.32 [0.12,0.89]**	**0.021**	0.54 [0.17,1.70]	0.295
Genotypes	CT and TT	117 (24.6)	156 (29.7)	0.77 [0.58,1.02]	0.071	0.81 [0.58,1.13]	0.221
Alleles	T	0.128	0.163				
VEGF	GG	162 (34.0)	200 (38.0)	1.00		1.00	
634G/C	GC	232 (48.8)	248 (47.2)	1.16 [0.88,1.52]	0.302	1.10 [0.79,1.53]	0.583
	CC	82 (17.2)	78 (14.8)	1.30 [0.89,1.88]	0.170	1.13 [0.73,1.75]	0.590
Genotypes	GC and CC	314 (66.0)	326 (62.0)	1.19 [0.92,1.54]	0.189	1.11 [0.81,1.51]	0.516
Alleles	C	0.416	0.384				
VEGF	GG	254 (53.4)	296 (56.3)	1.00		1.00	
1612G/A	GA	188 (39.5)	198 (37.6)	1.11 [0.85,1.44]	0.447	1.22 [0.90,1.67]	0.203
	AA	34 (7.1)	32 (6.1)	1.24 [0.74,2.06]	0.412	1.16 [0.64,2.11]	0.628
Genotypes	GA and AA	222 (46.6)	230 (43.7)	1.13 [0.88,1.44]	0.355	1.21 [0.90,1.63]	0.209
Alleles	A	0.269	0.249				
VEGF	CC	301 (63.2)	290 (55.1)	1.00		1.00	
2578C/A	CA	157 (33.0)	202 (38.4)	0.75 [0.58,0.98]	0.031	0.72 [0.53,0.98]	0.041
	AA	18 (3.8)	34 (6.5)	**0.51 [0.28,0.92]**	**0.024**	**0.48 [0.24,0.96]**	**0.038**
Genotypes	CA and AA	175 (36.8)	236 (44.9)	**0.71 [0.55,0.92]**	**0.009**	**0.69 [0.51,0.93]**	**0.014**
Alleles	A	0.203	0.257				

a *P* value for crude odds ratio (OR) and 95% confidence interval (CI).

b Adjusted for age, gender, HBV carrier state, family history of cancer, smoking, and drinking status.

c *P* value for adjusted odds ratio (OR) and 95% confidence interval (CI).

d Values in parentheses indicate percentages.

We further divided the data into subgroups based on age, sex, smoking status, alcohol drinking status, HBV carrier state and family history of cancer. We evaluated the HCC risk for each subgroup by estimating the ORs associated with the combined VEGF -2578 CA/AA variant genotypes compared with the VEGF -2578 CC genotypes, with adjustment for the aforementioned variables (Table 2). When stratifying by age, we found that the VEGF -2578 CA/AA variant genotypes was associated with a significantly risk of HCC among both older people (≥ 55 years old) and younger subjects (< 55 years old). Similarly, When stratifying by HBV carrier state, we found that the VEGF -2578 CA/AA variant genotype was associated with a significantly risk of HCC among both HBsAg-positive individuals and HBsAg-negative people. However, in the subgroup analyses by sex, smoking status, alcohol drinking status and family history of cancer, the risk associated with the combined CA/AA variant genotype was more evident for men (adjusted OR = 0.43, 95% CI = 0.29–0.63), never-smokers (adjusted OR = 0.57, 95% CI = 0.38–0.87), never-drinkers (adjusted OR = 0.57, 95% CI = 0.40–0.82), and subjects without family history of cancer (adjusted OR = 0.56, 95% CI = 0.41–0.77). No significant association was observed between the other three polymorphisms of the *VEGF* gene and risk of HCC in any stratification analysis (data not shown).

**Table 2:** Associations and stratification analysis of VEGFA 2578 C/A polymorphism and HCC risk

Variable	Genotypes	HCC (%) *n* = 476	Control (%) *n* = 526	Adjusted OR (95% CI)	*P* value
Age					
< 55 years	CC	169 (62.6)	175 (55.9)	1.00	
	CA + AA	101 (37.4)	138 (44.1)	0.57 (0.38,0.87)	0.009
≥ 55 years	CC	132 (64.1)	115 (54.0)	1.00	
	CA + AA	74 (35.9)	98 (46.0)	0.53 (0.33,0.84)	0.007
Gender					
Female	CC	76 (61.3)	87 (62.1)	1.00	
	CA + AA	48 (38.7)	53 (37.9)	1.06 (0.60,1.88)	0.840
Male	CC	225 (63.9)	203 (52.6)	1.00	
	CA + AA	127 (36.1)	183 (47.4)	0.43 (0.29,0.63)	0.000
Smoking					
Yes	CC	159 (62.1)	97 (54.5)	1.00	
	CA + AA	97 (37.9)	81 (45.5)	0.61 (0.38,1.00)	0.054
No	CC	142 (64.5)	193 (55.5)	1.00	
	CA + AA	78 (35.5)	155 (44.5)	0.57 (0.38,0.87)	0.008
Drinking					
Yes	CC	79 (60.8)	71 (57.7)	1.00	
	CA + AA	51 (39.2)	52 (42.3)	0.61 (0.32,1.18)	0.143
No	CC	222 (64.2)	219 (54.3)	1.00	
	CA + AA	124 (35.8)	184 (45.7)	0.57 (0.40,0.82)	0.002
HBV					
HbsAg (+)	CC	194 (60.2)	37 (43.5)	1.00	
	CA + AA	128 (39.8)	48 (56.5)	0.53 (0.32,0.86)	0.011
HbsAg (–)	CC	107 (69.5)	253 (57.4)	1.00	
	CA + AA	47 (30.5)	188 (42.6)	0.60 (0.40,0.89)	0.010
Family history of cancer					
Yes	CC	29 (64.4)	24 (51.1)	1.00	
	CA + AA	16 (35.6)	23 (48.9)	0.48 (0.16,1.46)	0.194
No	CC	272 (63.1)	266 (55.5)	1.00	
	CA + AA	159 (36.9)	213 (44.5)	0.56 (0.41,0.77)	0.000

### Genotype effects on postoperative recurrence of resected HCC

We also investigated the association between VEGF SNPs and postoperative recurrence of resected HCC. As shown in Table 3, we found that only the VEGF -2578 C>A polymorphism was significantly associated with the postoperative recurrence of HCC. The variant genotypes showed a lower recurrence rate than that of the wild genotype (OR_AC+AA vs. CC_, 0.51; 95% CI, 0.29–0.88; *P* = 0.017).

**Table 3:** SNPs of VEGFA genes and postoperative recurrence of resected HCC

SNP	Genotype	Recur (+), *n* (%)	Recur (−), *n* (%)	Adjusted OR^†^	95% CI	*p* value
VEGF	GG	42 (38.5)	37 (32.7)	Ref		
634G/C	GC/CC	67 (61.5)	76 (67.3)	0.78	0.45–1.37	0.395
VEGF	CC	82 (75.2)	86 (76.1)	Ref		
936 C/T	CT/TT	27 (24.8)	27 (23.9)	1.06	0.57–1.99	0.846
VEGF	GG	64 (58.7)	61 (54.0)	Ref		
1612G/A	GA/AA	45 (41.3)	52 (46.0)	0.89	0.52–1.54	0.675
VEGF	CC	72 (66.1)	57 (50.4)	Ref		
2578C/A	CA/AA	37 (33.9)	56 (49.6)	0.51	0.29–0.88	0.017

SNP, single nucleotide polymorphism; HCC, hepatocelluar carcinoma; Recur, postoperative recurrence; OR, odds ratio; CI, confidence interval.

† Adjusted for age, gender, HBV carrier state, family history of cancer, smoking, and drinking status.

### Genotype effects on overall survival of resected HCC

**Figure 1:**
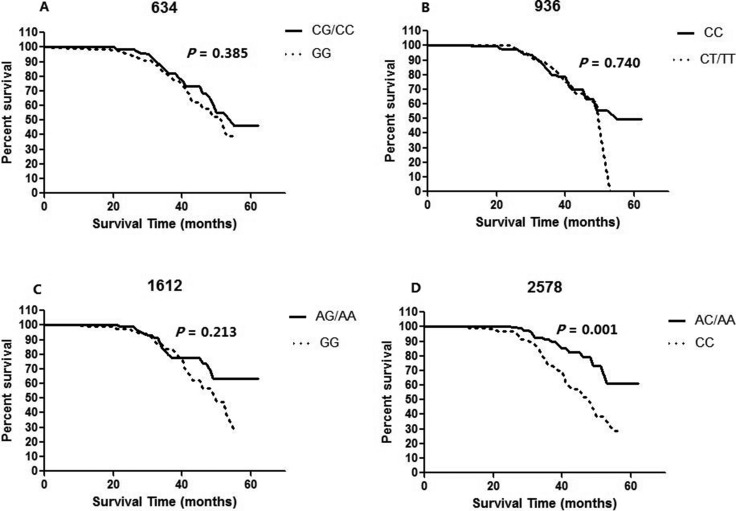
Kaplan-Meier curves of overall survival of curative resected HCC with each (**A**) 634 genotype, (**B**) 936 genotype, (**C**) 1612 genotype and (**D**) 2578 genotype of the VEGF gene. *P* value was calculated using a log-rank test.

In order to eliminate the influence of different treatment methods to HCC prognosis, we detected the SNPs genotype effects on OS of curative resected HCC. There were 52 deaths (23.4%) among the 222 resected HCC cases and the median survival time (MST) of these cases was 53 months in the cohort patients. By the Kaplan-Meier analysis, a tumor size larger than 5 cm, the diffuse type of HCC, advanced tumor staging (both TNM III/IV stages and BCLC B/C/D stages), the presence of portal vein invasion, lymph node metastasis, microvascular invasion, and microsatellite nodule, and advanced edmondson grade (III and IV) were factors that were significantly associated with reduced OS (Supplementary Table 5). Table 4 and Figure 1 shows the association between VEGF VEGF -936C>T, -634G>C, -1612 G/A and -2578 C>A genotypes and OS of the resected HCC patients. Of the 4 SNPs evaluated in the VEGF gene, we found that only the VEGF -2578 C>A polymorphism was significantly associated with the survival of the resected HCC patients (AA/AC vs. CC, *P* = 0.001).

**Table 4:** Overall survival by VEGFA genotype in patients with resected HCC

Genotype	Cases, *n*	Deaths, *n*	MST (Mo)	95% CI (Mo)	*P* value
VEGF 634G/C*					0.385
GG	79	23	53	43.4–62.6	
GC/CC	143	29	51	48.4–54.6	
VEGF 936 C/T*					0.740
CC	168	37	51	48.3–54.0	
CT/TT	54	15	50	46.5–53.4	
VEGF 1612G/A*					0.213
GG	125	34	50	43.9–56.1	
GA/AA	97	18	53	49.8–56.8	
VEGF 2578C/A*					0.001
CC	129	38	48	41.7–54.3	
CA/AA	93	14	55	51.7–58.4	

HCC, hepatocelluar carcinoma; MST, median survival time; Mo, months; CI, confidence interval.

* Mean survival time was provided when MST could not be calculated.

**Figure 2:**
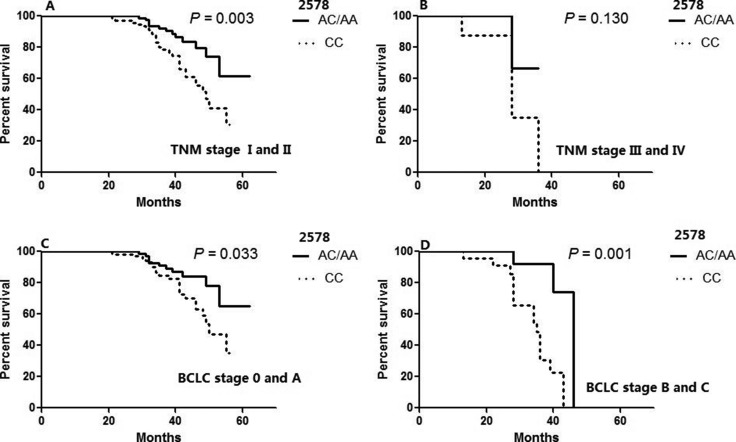
Overall survival of patients with resected HCC according to VEGF 2578 genotypes in different tumor stages (**A**) TNM stage I and II (**B**) TNM stage III and IV (**C**) BCLC stage 0 and A (**D**) BCLC stage B and C. *P* value was calculated using a log-rank test.

Furthermore, we also conducted a multivariate analysis of the effects of genotype on survival using Cox proportional hazards models adjusted for other important clinical factors which were proved to be associated with OS in the univariate analysis. We re-evaluated the associations for each -936C>T, -634G>C, -1612 G/A and -2578 C>A following adjustments for the clinical characteristics. As shown in Table 5, the -2578 C>A remained as significant independent predictors of survival among HCC patients (AA/AC vs. CC; adjusted HR = 0.27, 95% CI = 0.13–0.52). Moreover, among the important clinical factors, only the diffuse type of HCC, the presence of portal vein invasion, microvascular invasion, and advanced edmondson grade (III and IV) remained as significant independent predictors of reduced survival. In addition, we stratified the OS of patients according to VEGF 2578 genotypes by different tumor stages which were represented in Figure 2. The predictive power of genotype for survival was statistically apparent except for patients with TNM stage III and IV.

**Table 5:** Cox multivariate regression analysis of potential factors for overall survival in patients with resected HCC

Variables	Adjusted HR*	(95% CI)*	P*
Tumor size (> 5 cm vs. ≤ 5 cm)	1.099	0.364–3.318	0.867
Tumor type (diffuse vs. nodular)	3.887	1.861–8.122	0.000
Portal vein invasion (yes vs.no)	8.622	1.403–52.999	0.020
Lymph node metastasis (yes vs.no)	3.566	0.610–20.836	0.158
Microvascular invasion (yes vs.no)	3.471	1.628–7.401	0.001
Microsatellite nodule (yes vs.no)	1.572	0.667–3.703	0.301
Edmondson grade (III/IV vs.I/II)	2.549	1.264–5.138	0.009
VEGFA 2578 C/A (AA/AC vs. CC)	0.265	0.134–0.524	0.000

* HR (95% confidence interval [CI]) and *P* values for the 2578 genotype for overall survival were adjusted according to important clinical characteristics.

### VEGF genotypes and VEGF expression levels

The median serum concentration of VEGF detected was 383.02 pg/ml (range 88.96–1200.37 pg/ml) in HCC patients (*n* = 60) and 146.89 pg/ml (range 45.01–646.33 pg/ml) in healthy control subjects (*n* = 60). The serum levels of VEGF detected in HCC patients were significantly higher than those in the healthy control subjects (*P* < 0.000). However, when studying the relationship between the VEGF polymorphisms and serum levels among HCC patients and healthy controls, we found no significant association between VEGF SNPs genotype and serum levels of VEGF.

## DISCUSSION

In this study, we investigated the association of VEGF -936C>T, -634G>C, -1612 G/A and -2578C>A polymorphisms with susceptibility and clinical outcome of HCC in a Chinese Han population. To the best of our knowledge, this is the first study that has investigated the association between SNPs of VEGF genes and the occurrence and clinical outcomes of HCC simultaneously. Our results showed that the VEGF -2578C>A polymorphism was significantly associated with HCC susceptibility among both HBsAg-positive and HBsAg- negative individuals at different ages. Despite the serum levels of VEGF detected in HCC patients were significantly higher than those in the healthy control subjects, we found no significant association between VEGF SNPs genotype and serum levels of VEGF. In addition, we demonstrated that the VEGF -2578C>A polymorphism was associated with significantly decreased postoperative recurrence and improved OS of resected HCC patients.

In our study, the polymorphism of VEGF -2578C>A was found to be associated with the development and the OS in resected HCC patients. The exact mechanism for the correlation needs further exploring because we found no significant association between VEGF SNPs genotype and serum levels of VEGF. However, previous published studies on the functions of the VEGF -2578C>A SNP together with their genetic variants may help us understand the potential roles of the polymorphism. The -2578C/A polymorphism, which was a functional SNP in VEGF gene promoter region, are associated with altered VEGF secretion [18]. Moreover, Shahbazi *et al* [32] reported that the genotype C/C of the SNP rs699947 in the -2578 nucleotide position of the VEGF promoter, had been associated with higher levels of VEGF in blood mononuclear cells. In addition, Poon *et al* [16] found that a high serum level of VEGF is a predictor of poor outcome of HCC. Therefore, it is biologically plausible that VEGF -2578C/A polymorphism was associated with a decreased HCC risk and the increased OS in resected HCC patients through a low serum level of VEGF caused by the 2578 C to A variant.

In recent years, the association between VEGF polymorphism and HCC risk has been investigated, however, the results are inconsistent. In 2010, He *et al* [27] firstly reported that an 18-bp insertion/deletion polymorphism (rs35569394) in the promoter region of VEGF gene was not associated with susceptibility to HCC in Chinese. However, Giacalone *et al* [28] found that carriers with the C allele of 936 C/T in the VEGF gene were more frequent in HCC versus liver cirrhosis and suggested that this SNP might predispose to the development of HCC. Moreover, Wu *et al* [15] demonstrated that two promoter SNPs (rs833061 and rs1570360) in VEGF were associated with susceptibility to HCC and the 936 C/T polymorphism was not associated with HCC. Our study found that the VEGF -2578C>A polymorphism was significantly associated with HCC and the 936 C/T polymorphism was not associated with HCC risk. The conflicting results could be attributable to the differences in demography, ethnicity, lifestyles, and other methodologic factors in the studies, such as small sample size, inadequate adjustment for confounding factors.

Alcohol consumption and cigarette smoking are important environmental risk factors for HCC [33, 34]. In addition, age, gender and family history of cancer might also affect the risk of HCC. Unlike Wu *et al*’s study [15], we also performed subgroup analyses by these risk factors. We found that the decrease in risk was more evident among men, never-smokers, never-drinkers, and subjects without family history of cancer for the VEGF -2578C>A polymorphism. Although the exact mechanism for the results of subgroup analyses were as yet unknown, some possibilities should be considered. Previous studies [33, 34] had proved that drinking and smoking were major risk factor for HCC. Especially in 2004, the International Agency for Research on Cancer had classified HCC as one of the tobacco-related cancers [35]. The environment risk factors, such as drinking and smoking, could increased the risk of HCC; while the VEGF -2578C/A polymorphism was associated a decreased risk of HCC. Therefore, the protect effect for HCC by the VEGF -2578C/A polymorphism may be eliminated by the environment risk factors after gene-environment interaction.

We also performed functional analysis for the VEGF SNPs to attempt to clarify the mechanism for the correlation between VEGF SNPs and risk and clinical outcome of HCC by measuring the serum VEGF levels among both HCC patients and healthy controls. Although the serum levels of VEGF detected in HCC patients were significantly higher than those in the healthy control subjects (*P* < 0.000), no significant association between VEGF SNPs genotype and serum levels of VEGF was observed. This may be because the simple size for measuring the serum VEGF levels was too small to detect the association between VEGF SNPs genotype and serum levels of VEGF. In addition, another explanation for the negative correlation was that the serum VEGF concentration could be regulated by many clinical factors and cytokines except for the VEGF gene polymorphism [29, 36].

In this study, several limitations need to be addressed. Firstly, these results should be interpreted with caution because the population only from Chinese Han, which reduces the possibility of confounding from ethnicity, but it does not permit extrapolation of the results to other ethnic groups because the allele frequency patterns of VEGF polymorphisms vary greatly between different ethnic groups. Secondly, the study sample size was relative small, especially for the association between VEGFA polymorphism and HCC prognosis.

In summary, this study investigated the association of VEGFA -936C>T, -634G>C, -1612 G/A and -2578C>A polymorphisms with risk and clinical outcome of HCC simultaneously in the same cohort patients and found the VEGFA -2578C>A polymorphism was associated with a significantly decreased HCC risk and improved OS of resected HCC patients. These results suggest that the VEGFA 2578 C/A polymorphism may play a potential role in the development and clinical outcome of HCC among Chinese Han population.

## MATERIALS AND METHODS

### Study population

Our population had been described previously [37]. Supplementally, identification of the stage of tumor is based on the tumor–node–metastasis (TNM) classification system which was promulgated by the American Joint Committee on Cancer and International Union Against Cancer in 2010 [38] and Barcelona-Clinic-Liver-Cancer (BCLC) stages [39]. Vascular invasion was recognized by the presence of thrombus adjacent to the tumor in portal and hepatic vein system with vague boundary confirmed by at least two imaging modalities [40]. Routine chest CT scan was performed to detect metastatic lesion(s). Bone metastasis of HCC was surveyed by bone scan and confirmed by MRI. Lymph node involvement was diagnosed by CT/MRI and/or postoperative pathology. Clinical information such as age, sex, tumor size, number of tumors, nodal invasion, metastasis, existence of vascular invasion, Child-Pugh classification, treatment modalities and the results of clinical laboratory tests were collected from the medical records. Based on Child-Pugh classification, the location of the tumor within the liver and modified TNM staging, we initially treated patients with one of four treatment modalities: liver resection, radiofrequency ablation, transarterial chemoe mboliz- ation, or conservative management (such as sorafenib). The overall survival time was calculated from the date of tumor resection or the first local treatment to the date of death or to the last follow-up. Informed consents were obtained according to the Declaration of Helsinki. A written informed consent was obtained from each subject involved in the study. The study was approved by the Ethics Committee of Sichuan University.

### Genotype analyses

The genomic DNA was isolated from whole blood samples using the whole blood DNA kit (Biotake corporation). The concentration of DNA was diluted to 20 ng/μL for working solutions and the isolated DNA was stored at −20°C. SNP genotyping was performed using MassARRAY system (Sequenom, San Diego, CA, USA) by means of matrix assisted laser desorption ionisation-time of flight mass spectrometry method (MALDI-TOF) according to the manufacturers instructions. Primers for PCR and single base extension were designed by using Assay Designer software package (Sequenom) (Supplementary Table 1). The detailed process for SNP genotyping could be found in elsewhere [37] and the Supplementary Table 2. For quality control, genotyping was performed without knowing the subjects’ status, and a 5% random samples were genotyped twice for each locus. To further validate the genotyping assay of MALDI-TOF for the four loci, about 5% of samples genotyped with MALDI-TOF were further confirmed by direct sequencing method. Among the 500 cases and 550 controls with DNA samples, the genotyping was successful for the four loci in 476 cases and 526 controls which were included in the final analyses.

### Serum VEGF levels

Blood samples were obtained from subjects with and without HCC, centrifuged and stored at −80°C until analysis. Commercial ELISA kit (R&D Systems, Minneapolis, MN, USA) was used according to the instructions of the manufacturer to measure VEGF-A concentrations in serum. Briefly, 100 μL serum samples and standards were added to a 96-well antibody-coated plate. Then the plate was washed three times with wash buffer. After then, the biotin conjugate-labeled second antibody was added in plate and stored at room temperature. One hour later the plate was washed again and the conjugate was added, sealed, shaken and stored at room temperature for 30 minutes and then washed three times again. The substrate was added in all plates and stored at room temperature out of light for 10 minutes and then the stop solution was added. The absorbance was measured at 450 nm.

### Statistical analysis

Hardy–Weinberg equilibrium (HWE) was tested by a goodness-of-fit *χ*^2^ test, to compare the observed genotype frequencies to the expected ones among the control subjects. The associations between VEGF genotypes and risk of HCC were estimated by computing the odds ratios (ORs) and their 95% confidence intervals (CIs) from both univariate and multivariate logistic regression analyses with adjustment for age, sex, HBV carrier state, alcohol intake, smoking status, and family history of cancer. Differences in VEGF serum levels among patients with HCC versus control subjects were examined using the Mann-Whitney *U* test. The relation of SNPs to the overall survival (OS) was identified using the Kaplan-Meier method with the log-rank test and Cox proportional hazard model. All statistical analyses were two sided and performed using SPSS version 16.0 for Windows statistical software (SPSS Inc., Chicago, IL, USA). A *P*-value of < 0.05 was considered as statistically significant.

## SUPPLEMENTARY FIGURE AND TABLES


