# Supplementary material for: Association of VEGFA polymorphisms with susceptibility and clinical outcome of hepatocellular carcinoma in a Chinese Han population
Source: Oncotarget. 2017 Jan 27;8(10):16488–97. doi: 10.18632/oncotarget.14870 (PMC5369979; doi:10.18632/oncotarget.14870)
Supplement: Supplementary file 1 [file oncotarget-08-16488-s001.pdf]

Association of VEGFA polymorphisms with susceptibility and clinical outcome of hepatocellular carcinoma in a Chinese Han population

Supplementary Materials

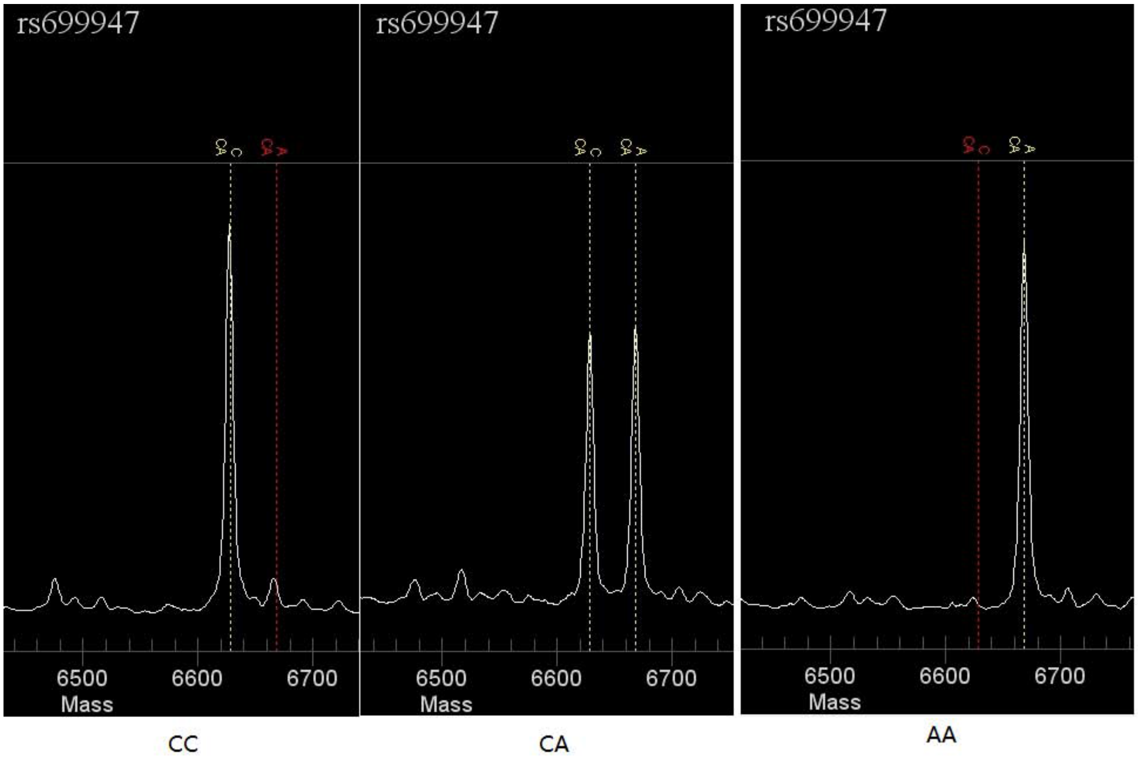

Supplementary Figure 1: Genotyping of VEGF 2578C/A polymorphism by MALDI-TOF. The horizontal axis represents the mass and the vertical axis represents the signal intensity. Around the 6600–6700 dalton in the horizontal axis, two lines (the C allele and the A allele) can be observed. If only a wave crest appeared at the C line, the genotype of this patient was CC (the left one); if only a wave crest appeared at the A line, the genotype of this patient was AA(the right one); if two wave crest appeared at both the C line and the A line, the genotype of this patient was CA (the middle one).

Supplementary Table 1: Primers for PCR and single base extension for genotyping VEGF polymorphisms

Polymorphism	Primers for PCR	Single base extension primers
936 C/T Rs3025039	F:ACGTTGGATGAGACTCCGGCGGAAGCATT<br>R:ACGTTGGATGATGGCGAATCCAATTCCAAG	CGGGCGGGTGACCCAGCA
634G/C Rs2010963	F:ACGTTGGATGAGAGAGAGCGCGCGGGCGT<br>R:ACGTTGGATGTCCGGCGGTCACCCCCAAAA	TGCGAGCAGCGAAAG
1612G/A Rs10434	F:ACGTTGGATGCCTTCGCTTACTCTCACCTG<br>R:ACGTTGGATGTTCTCTTCGCCGGGACATCT	CTGAGTTGCCCAGGAG
2578C/A Rs699947	F:ACGTTGGATGTTCTCAGTCCATGCCTCCAC<br>R:ACGTTGGATGAGTCAGTCTGATTATCCACC	GTCTGATTATCCACCCAGATC

Supplementary Table 2: The detailed process for SNP genotyping. See Supplementary_Table_2

Supplementary Table 3: Frequency distribution of baseline characteristics among HCC patients and controls

Variable	HCC (n = 476)	controls (n = 526)	OR (95% CI)	P-value
Age [n (%)]				0.372
< 55 years	270 (56.7)	313 (59.5)	1.00	
≥55 years	206 (43.3)	213 (40.5)	1.12 (0.87,1.44)	
Gender [n (%)]				0.839
Female	124 (26.1)	140 (26.6)	1.00	
Male	352 (73.9)	386 (73.4)	1.03 (0.78,1.36)	
Smoking status [n (%)]				< 0.001
Never	220 (46.2)	348 (66.2)	1.00	
Ever	256 (53.8)	178 (33.8)	2.28 (1.76,2.94)	
Drinking status [n (%)]				0.153
Never	346 (72.7)	403 (76.6)	1.00	
Ever	130 (27.3)	123 (23.4)	1.23 (0.93,1.64)	
HBV carrier state [n (%)]				< 0.001
HbsAg (−)	154 (32.4)	441 (83.8)	1.00	
HbsAg (+)	322 (67.6)	85 (16.2)	10.85 (8.03,14.66)	
Family history of cancer [n (%)]				0.777
No	431 (90.5)	479 (91.1)	1.00	
Yes	45 (9.5)	47 (8.9)	1.06 (0.69,1.63)	

Supplementary Table 4: Clinicopathologic characteristics of the enrolled patients with HCC

**Child-Pugh class *n* (%)**	
CH/A	366 (76.9)
B	79 (16.6)
C	31 (6.5)
Tumor size *n* (%)	
≤ 5 cm	240 (50.4)
> 5 cm	236 (49.6)
Tumor type, *n* (%)	
Single	291 (61.1)
Multiple	117 (24.6)
Diffuse	68 (14.3)
Tumor staging, *n* (%)	
Very early (0)	29 (6.1)
Early (A)	182 (38.2)
Intermediate (B)	90 (18.9)
Advanced (C)	144 (30.3)
Terminal (D)	31 (6.5)
Surgical resection *n* (%)	
No	254 (53.4)
Yes	222 (46.6)
Distant metastasis *n* (%)	
No	429 (90.1)
Yes	47 (9.9)
Vascular invasiona *n* (%)	
No	387 (81.3)
Yes	89 (18.7)
Lymph node metastasis *n* (%)	
No	384 (80.7)
Yes	92 (19.3)
AFP *n* (%)	
> 200 ng/ml	205 (43.1)
≤ 200 ng/ml	271 (56.9)
Liver cirrhosis *n* (%)	
Absent	155 (32.6)
Present	321 (67.4)

[a]Portal vein or Hepaticvein invasion according to radiology.

Supplementary Table 5: Overall survival according to clinicopathologic features of resected HCC

Characteristics	Patients, *n*	Deaths, *n*	MST (Mo)	95% CI (Mo)	*P* value
Age, years					0.243
≤ 55	118	33	50	45.8–54.1	
> 55	104	19	52*	48.9–56.2	
Gender					0.628
Male	161	38	53	46.9–59.1	
Female	61	14	49*	45.9–51.8	
Smoking					0.751
Yes	116	29	50*	47.0–53.7	
No	106	23	53	44.8–61.2	
Drinking					0.195
Yes	64	19	55	35.2–74.7	
No	158	33	53	47.6–58.3	
HBsAg					0.762
Positive	167	38	55	†	
Negative	55	14	53	45.8–60.2	
AFP level (ng/mL)					0.854
≤ 200	137	33	55	46.8–63.1	
> 200	85	19	49	†	
Child-Pugh Class					NA
CH/A	222	52	53	46.6–59.3	
B/C	0	0	♂	†	
Tumor size (cm)					0.001
≤ 5	203	44	55	†	
> 5	19	8	36	25.1–46.8	
Tumor type					< 0.001
Single	189	35	55	†	
Multiple	33	17	39	34.4–43.5	
Portal vein invasion					0.011
Yes	4	2	36	1.5–70.4	
No	218	50	53	46.6–59.3	
Tumor BCLC staging					< 0.001
0 and A	185	34	55	†	
B to C	37	18	39	35.1–42.8	
Tumor TNM staging					< 0.001
I or II	210	45	55	47.2–62.7	
III or IV	12	7	28	18.1–37.8	
Distant metastasis					NA
Yes	0	0	♂	†	
No	222	52	53	46.6–59.3	
Edmondson grade					0.037
I or II	174	38	53	†	
III or IV	48	14	45*	40.9–49.8	
Microvascular invasion					< 0.001
Present	24	11	35	26.7–43.3	
Absent	198	41	55	47.1–62.8	
Microsatellite nodule					0.017
Present	31	11	40*	37.0–44.8	
Absent	191	41	53	46.6–59.3	
Background cirrhosis					0.851
Present	157	34	49	43.7–54.3	
Absent	65	18	55	†	
Lymph node metastasis					< 0.001
Present	5	2	28	†	
Absent	217	50	53	46.6–59.3	

HCC, hepatocelluar carcinoma; MST, median survival time; Mo, months; AFP, alpha-fetoprotein; NA, not applicable.
*Mean survival time was provided when MST could not be calculated.
†95% CI could not calculated.
♂Both Mean survival time and MST could not be calculated.
